# On the essentiality of the angiotensin converting enzyme 2 receptor for SARS‐CoV‐2 infection and the potential of soluble angiotensin converting enzyme 2 proteins as universal approach for variants causing COVID‐19

**DOI:** 10.1002/ctm2.1080

**Published:** 2022-10-13

**Authors:** Luise Hassler, Josef Penninger, Daniel Batlle

**Affiliations:** ^1^ Division of Nephrology/Hypertension Department of Medicine Northwestern University Feinberg School of Medicine Chicago Illinois USA; ^2^ Institute of Molecular Biotechnology of the Austrian Academy of Sciences (IMBA) Vienna Austria; ^3^ Department of Medical Genetics Life Sciences Institute University of British Columbia Vancouver BC Canada

1

After the outbreak of severe actute respiratory syndrom coronavirus (SARS‐CoV) in 2003, it was shown that angiotensin converting enzyme 2 (ACE2) was the receptor for this coronavrius.^1^ As early as January 2020, it was reported that ACE2 was also the main cell entry receptor for SARS‐CoV‐2.^2^ There have been various reports on additional potential receptors for SARS‐CoV‐2 cell entry.^3^, ^4^, ^5^ The importance of these additional proteins for SARS‐CoV‐2 cell entry, however, remains unclear. Here, we want to emphasize the evidence in favour of the essentiality of ACE2 for SARS‐CoV‐2 infection. Normally, human organoids, like human and monkey cell lines, can be easily infected with SARS‐CoV‐2 when they expre  both ACE2 as well as TMPRSS2, one of the proteases critical for the activation of the SARS‐CoV‐2–ACE2 complex prior to internalization.^6^, ^7^ We have provided evidence that kidney organoids derived from cell lines that lack ACE2 could not be infected with SARS‐CoV‐2 as judged by lack of nucleoprotein staining and ACE2 mRNA and protein levels after exposure to a high dose of SARS‐CoV‐2.^8^ Further evidence in vivo supports the essentiality of ACE2 for SARS‐CoV‐2 infectivity. Using ACE2‐deficient mice inoculated with a mouse‐adapted SARS‐CoV‐2 variant, it was shown that these mice were not susceptible to SARS‐CoV‐2 infection.^9^ This was in sharp contrast with wild‐type mice that rapidly lost weight and developed severe pneumonia after viral inoculation.^9^ Altogether, these two findings provide strong direct evidence that SARS‐CoV‐2 infection can only occur in the presence of membrane bound ACE2. The essentiality of membrane bound ACE2 for SARS‐CoV‐2 infectivity forms the basis for the design of therapeutic modalities to combat SARS‐CoV‐2 related infections.

Early in 2020, before COVID‐19 was declared a pandemic, our groups independently proposed that administering soluble ACE2 proteins would be useful to combat COVID‐19.^10^, ^11^ ACE2 exists in two forms, a full‐length membrane bound form, and a soluble form that lacks the transmembrane domain necessary for anchoring in the cell membrane^12^ that circulates in the blood in small amounts.^13^ Both forms of ACE2 are enzymatically active and contain the amino acid sequence used by the receptor binding domain of the SARS‐CoV‐2 spike protein.^12^ Soluble ACE2, by acting as a decoy, can intercept SARS‐CoV‐2 from binding to membrane bound ACE2. The rationale for the decoy hypothesis came from earlier work showing the inhibition of SARS‐CoV cell entry into Vero E6 cells and 293T cells with soluble ACE2.^1^, ^14^ The hypothesis was confirmed in human organoids by Monteil et al. and later by Wysocki et al.^6^, ^7^ Both studies showed that soluble ACE2 proteins effectively neutralised SARS‐CoV‐2 in human kidney organoids and that high concentrations were required.^6^, ^7^ The only clinical grade version of a soluble ACE2 protein available (APN01) was administered to one patient hospitalised with severe COVID‐19.^15^ The patient was treated with soluble ACE2 intravenous infusion (0.4 mg/kg body weight) for 5 min, twice daily for 7 days. The administration of APN01 was well‐tolerated without obvious drug‐related side effects and Ang II levels declined rapidly after the first dose.^15^ The administration of this soluble ACE2 protein, moreover did not interfere with development of neutralising antibodies. Real‐time polymerase chain reaction revealed an increase of tracheal aspirate SARS‐CoV‐2 that was cleared over the treatment period.^15^ In a phase 2 study (NCT04335136), APN01 was administered to COVID‐19 patients intravenously twice daily and failed to meet the primary outcomes (all cause‐death or invasive mechanical ventilation up to 28 days or hospital discharge). Since then, a nebulized version of APN01 that was shown to be safe for administration via inhaler^16^ is currently being tested in a phase 1 study (NCT05065645). Later on, soluble ACE2 proteins have been bioengineered as well. A protein bioengineered to have prolonged duration of action by using an albumin binding domain (ABD), and to have increased binding affinity to SARS‐CoV‐2 by dimerizing it via a dodecapeptide motif (DDC), termed ACE2 618‐DDC–ABD, protected k18hACE2 mice from lethal SARS‐CoV‐2 infection.^17^ The high efficacy was likely due to the intranasal administration combined with systemic administration at high doses. Viral titers were reduced or non‐detectable and lung histopathology was markedly improved in this mouse model.^17^ In a more recent study using the same mouse model, the comparison of intranasal to systemic administration of ACE2 618‐DDC–ABD showed that intranasal is superior to systemic administration to improve mortality and morbidity.^18^ An additional potential therapeutic benefit of ACE2 proteins is their enzymatic activity. ACE2 is a monocarboxypeptidase that converts Angiotensin II to form Angiotensin (1‐7).^12^, ^13^ This is a favourable feature particularly in SARS‐CoV‐2 associated lung injury, where ACE2 is depleted due to internalization of the SARS‐CoV‐2–ACE2‐complex.^19^, ^20^ Subsequently, the excessive Angiotensin II levels worsen lung injury.^19^, ^20^ By administering exogenous soluble ACE2 that is enzymatically active, the excessive Angiotensin II can be metabolised to form Angiotensin (1‐7). While the main component of the therapeutic action of soluble ACE2 proteins is the decoy effect, the provision of enzymatic activity, particularly with ongoing membrane bound ACE2 depletion, can only add to their therapeutic efficacy.

In this perspective, we want to emphasise the merits of soluble ACE2 proteins as universal approach to combat different variants of SARS‐CoV‐2 and future emerging coronaviruses that use ACE2 as their main cell entry receptor. Studies in Vero E6 cells, which are permissive for SARS‐CoV‐2 infection, revealed that several SARS‐CoV‐2 variants including omicron can all be neutralised by soluble ACE2.^21^ The soluble ACE2 protein, termed ACE2 618‐DDC–ABD, likewise, also displayed the neutralising effect against several variants of SARS‐CoV‐2.^17^ This universal action offers potentially important therapeutic advantages as compared to monoclonal antibodies. It has been shown that monoclonal antibodies and also vaccine elicited antibodies escape immunization, especially for the most recent variant of concern, omicron.^22^, ^23^ Even though these variants of concern carry mutations that lead to immune escape, the decoy effect of soluble ACE2 still remains effective. Multiple passaging of SARS‐CoV‐2 in the presence of soluble ACE2, moreover, has been shown to not induce any escape mutations which is in contrast to multiple passaging in the presence of monoclonal antibodies.^24^ We surmise that if administered at the right time and preferably intranasally, soluble ACE2 proteins offer protection for all SARS‐CoV‐2 variants and future emerging coronaviruses and offer great therapeutic potential.

## CONFLICT OF INTEREST

D.B. is coinventor of patents entitled “Active Low Molecular Weight Variants of Angiotensin Converting Enzyme 2,” “Active low molecular weight variants of Angiotensin Converting Enzyme 2 (ACE2) for the treatment of diseases and conditions of the eye,” and “Soluble ACE2 Variants and Uses Therefor,” which includes the use of prevention and treatment of COVID‐19. D.B. is the founder of Angiotensin Therapeutics Inc. D.B. has received a grant and consulting fees from AstraZeneca unrelated to this work. J.M.P. is shareholder of Apeiron Biologics that is developing soluble ACE2 for COVID‐19 therapy.
